# Geospatial distribution and machine learning algorithms for assessing water quality in surface water bodies of Morocco

**DOI:** 10.1038/s41598-023-47991-z

**Published:** 2023-11-23

**Authors:** Rachida El Morabet, Larbi Barhazi, Soufiane Bouhafa, Mohammed Abdullah Dahim, Roohul Abad Khan, Nadeem A. Khan

**Affiliations:** 1 LADES Lab FLSH-M, Department of Geography, Hassan II University of Casablanca, Mohammedia, Morocco; 2 Department of Civil Engineering, King Khalid University, Abha, Saudi Arabia; 3 Interdisciplinary Research Center for Membranes and Water Security (IRC-MWS), King Fahd University of Petroleum and Minerals, Dammam, Saudi Arabia

**Keywords:** Environmental chemistry, Environmental impact, Environmental sciences, Environmental social sciences

## Abstract

Surface waterbodies being primary source of water for human consumption are being investigated for its quality globally. This study evaluated water quality in three rivers (River Nfifikh, Hassar and El Maleh) of Mohammedia prefecture, Morocco in terms of heavy metals occurrence during two seasons of winter and spring. The heavy metals analyzed were cadmium, iron, copper, zinc, and lead. Heavy metal pollution index was derived to quantify water quality and pollution. Hazard quotient and carcinogenic risk were calculated to determine possible health risk. Modelling and prediction were performed using random forest, support vector machine and artificial neural network. The heavy metal concentration was lower in the winter season than in the spring season. Heavy metal pollution index (H.P.I.) was in the range of 1.5–2 during the winter season and 2–3 during the spring season. In the Nfifikh river, Cd^2+^ and Fe were the main polluting heavy metal. H.Q. was < 1 in all three rivers, which signified no adverse health effect from exposure to heavy metals. However, carcinogenic risk assessment revealed that 1 in every 100 people was susceptible to cancer during the life span of 70 years. Based on the control point reference, it was found that Mohammedia prefecture as river water was already contaminated before it entered the prefecture boundary. This was again validated with the water lagoon Douar El Marja which is located near the industrial zones of Mohammedia prefecture. Future studies are required to investigate pollution of rivers prior to their entry in Mohammedia prefecture to identify potential source and adopt mitigation measures accordingly.

## Introduction

Surface water sources have been the foundation of urban development since ancient times as they serve as renewable water sources throughout the year^1^. Agriculture development to support the food supply of the existing population elevates the importance of surface water bodies^2^. This dependency on surface water sources is undeniable globally. However, economic development (urbanization and industrialization) have led to deterioration of these precious water resources^3,4^. River waters have been reported for pollution, and the concern is only increasing with heavy metal contamination^5^. Pollution from heavy metal has been in focus in the last couple of decades^6^. Heavy metal occurrence in water resources is attributed to natural and anthropogenic activities^7^. However, natural incidences of heavy metal contamination are rare and pose no serious threat^5^. Anthropogenic activities are primary responsible for water resources contamination^8^.

Highly toxic nature and adverse impact on human health from heavy metals have raised concerns globally^9,10^. Heavy metals non-biodegradability, persistence characteristics, and bio-accumulation properties pose risk to human health and aquatic life^11^. Recent studies of river water quality assessment regarding heavy metals have not been in focus in Morocco. Fez river urban catchment area modelling was done by Bouizrou et al.^12^. Berger et al.^13^ investigated interrelationship between social and ecological aspect of Draa river in southern Morocco. Abba et al.^14^ investigated the river, El Kell, in physicochemical and bacteriological parameters. Barakat et al. analyzed River Er Rabia water quality regarding physicochemical parameters. Perrin et al.^15^ investigated river Fez and Sebou in physicochemical, nutrients and chromium concentration. Nader lagoon environmental state was analyzed in water, sediment and microfauna by Ruiz et al.^16^. The geoenvironmental risk was assessed using the G.I.S. tool in the upper catchment area of river Sebou by De Waele et al.^17^. Biomarkers were used by Benyaich et al.^18^ for water quality assessment of river Souss estuary, bay of Agadir. Based on these studies it can be inferred that investigation of river water in Morocco, in terms of heavy metals is still lacking. Additionally, the toxic risk posed to ecosystem from heavy metal is of concern which necessitates investigation of water resources in terms of heavy metal occurrence. Also, prediction and modelling of water quality is gaining focus in recent research works. Kantipudi et al. has developed hyperspectral remote sensor data based model for analysis of water quality. El Bilali et al.^19^ have used machine learning algorithms for forecasting groundwater quality. van Wijnen et al.^20^ had modelled rivers transportation globally for microplastic using GREMiS model. Nonetheless, modelling prediction of water quality is still yet to explored to its potential. However, the literature on forecasting or modelling water quality in terms of groundwater and surface water is still lacking. This again calls for studies forecasting.

El Maleh, Hassar and Nfifikh rivers pass through Mohammedia prefecture and are primary source of water for human consumption, animal husbandry and agricultural activities^21^. Also, despite many rivers of Morocco being reported for their water quality based on physicochemical parameters and biological indicators, River El Maleh, Hassar and Nfifikh have yet to be investigated. This study was conducted to address the above-mentioned research gaps in terms of surface water quality assessment based on heavy metal occurrence and its potential forecasting. Hence, this study was carried out to address the abovesaid research gaps. The main objective of the study is to assess the performance of machine learning algorithm in predicting water quality of surface water bodies in Mohammedia prefecture, Morocco. This approach renders the study as first of its kind the region. The study also investigated heavy metal occurrence in river Hassar, El Maleh and Nfifikh, impact of season on water quality and estimated potential health risk from heavy metal occurrence. The study is limited to only Mohammedia prefecture of Morocco. The analysis is based on only surface water sources. The study is based on only five heavy metals.

## Materials and method

### Study area

**Figure 1:**
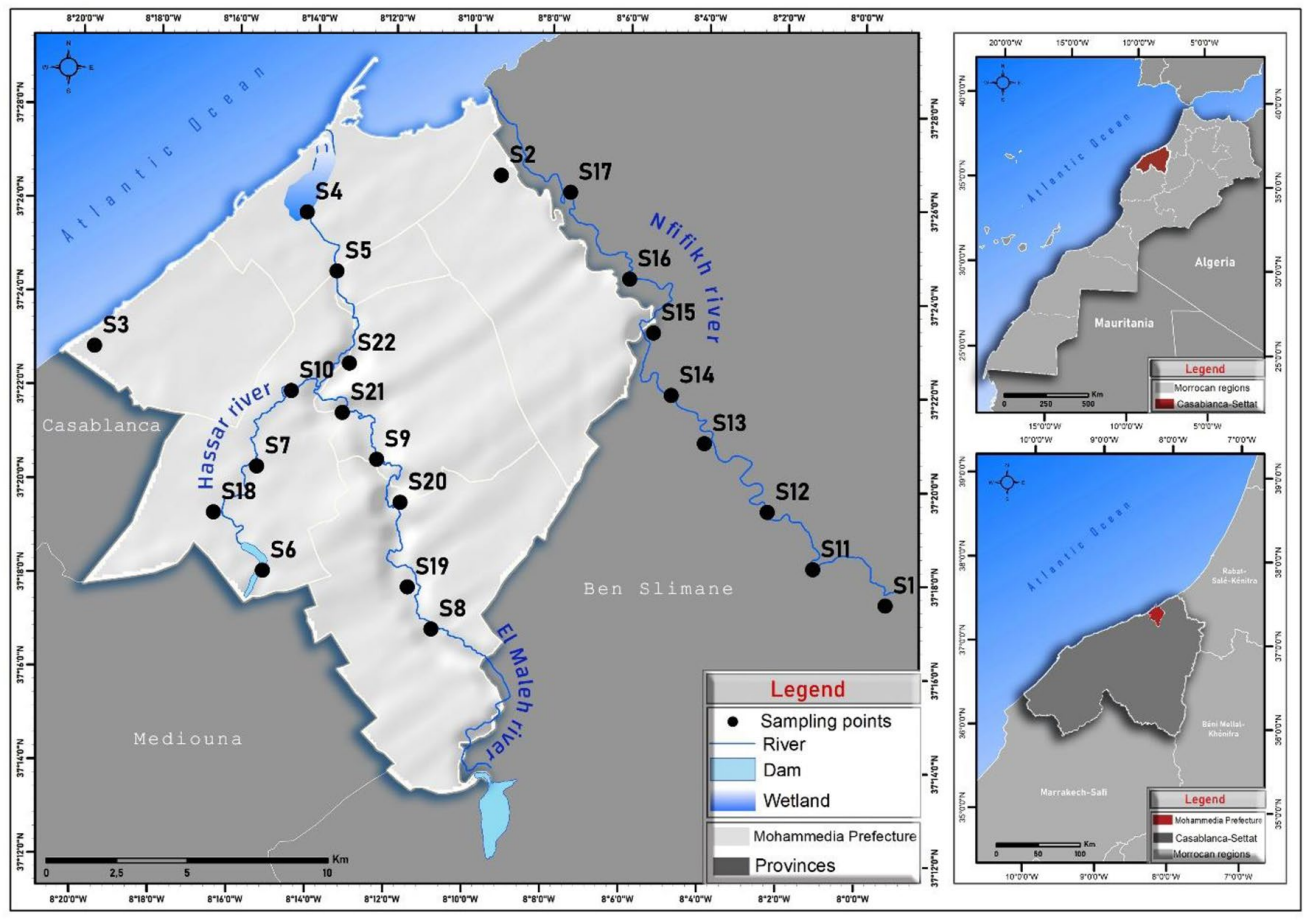
The study area of Mohammedia prefecture, Casablanca-Setat region, Morocco depicting sample points along the three rivers.

Mohammedia prefecture lies in the Casablanca-Setat region, Morocco. The warm months in Mohammedia experiences temperature in range of 27 °C and 20 °C. the cooler months November to March experiences on average temperature range of 11 °C to 18 °C. Starting from September to May Mohammedia prefecture experiences rainfall with average of 8-11 mm. Three rivers viz., El Maleh, Hassar and Nfifikh flow in Mohammedia prefecture^22^. El Maleh river flows through the heart of Mohammedia prefecture. Hassar is a tributary of the El Maleh river originating from south of the prefecture and meets El Maleh river in almost the centre of the prefecture. Nfifikh runs on the western border of Mohammedia prefecture. The prefecture experiences arid to semi-arid climatic conditions attributed to the Atlantic Ocean on the North and East of the prefecture region^23^. Mohammedia prefecture is host to the port city of Mohammedia. Being a port city, it is also host to various industries. Besides the Urban area, Mohammedia prefecture exhibits intensive agricultural activities. Figure 1 presents the study area and the three rivers being investigated in this study. The maps used in this study were generated by authors using ArcGIS software with version 10.7.1.

### Surface water sampling collection

To assess impact of urban interference in river water quality a total of 9 samples were collected. The samples were obtained to assess river water quality before entering Mohammedia prefecture, then before and after the Urban landscape of Mohammedia city. This also aided in identifying impact of agricultural landscape on the river water quality before it reaches Urban landscape. Also, another existing surface water body was a coastal lagoon was investigated represented as Sample point, S3 is a lagoon situated on the coastal area of the Atlantic Ocean. While sample S2 of Nfifikh river was the only point as Nfifikh river runs eastern border of Mohammedia. In comparison, point S2 was selected to evaluate the impact of Mohammedia prefecture on river Nfifikh. Other 7 points were selected for El Maleh and Hassar river. Two sample points S6 and S7 on Hassar river were selected to determine their water quality. Point S10 was selected where Hassar river merges with El Maleh river. Points S8 and S9 were selected to assess compare water quality for Hassar river and El Maleh river concerning point S10. Points S4 and S5 were chosen to determine the impact of Mohammedia city on river water quality. River samples were collected during the winter (January 2023) and spring (April 2023). Three replicate samples were obtained for each sample point.

Water samples were obtained at depth of 20 cm to avoid possible water surface layer interference^24^. Brown bottles were used for water sampling to inhibit light interference^25^. Collected water samples were stored at 4 °C to 10 °C temperature range in ice cooler, before transporting to Laboratory. In Laboratory, if the samples were not immediately tested, they were again stored at 4 °C in the refrigerator^5^. Before testing, the samples were left to achieve room temperature of 24 °C ± 2 °C^26^.

### Instrumental testing

River water samples were analyzed for heavy metals occurrence. Heavy metals covered in this study are cadmium (Cd^2+^), copper (Cu), iron (Fe), lead (Pb) and zinc (Zn). These heavy metals are also commonly analyzed in other research works^21,27,28^. The current study is restricted to only five heavy metals which were based on literature and local water monitoring agencies. Since, this study is first of its kind. Its scope is limited. This study can be reference for future studies which may incorporate other heavy metals viz. arsenic, nickel, chromium etc. for investigation. The sample testing was conducted in African Laboratory for Mining and Environment. UNICAM 929 AA solar atomic absorption spectrophotometer was used for testing heavy metal concentration in the river water sample. The instrument was calibrated after every 15 samples^29^. The accuracy was again validated against blank samples with double distilled water. Also, internal standards were used for accuracy obtained from sigma Aldrich. Glassware used in the analysis was soaked in HNO_3_ and then rinsed with deionized water before testing samples^30^.

### Heavy metal pollution index (HPI)

The indexing approach for analysis of water quality has been adopted for simple presentation and a better understanding of individual parameters that affect water quality. The indexing approach in most studies comprises either proportional weightage concerning permissible standards of each parameter^5,25,31,32^ or assigning weightage to each parameter on a fixed scale^33^. However, the fixed scale method has one advantage: it refines the weightage of each parameter again concerning the total weightage of all parameters under consideration. This study has adopted the combined approach of both approaches. The indexing approach used in this study in combination of the approaches used in the above-mentioned literature. First, obtaining relative weight (Rw) of each heavy metal with respect to its permissible limits as per Eq. (1). Table S3 provides the Rw for each heavy meatal analysed in this study. The relative weight was divided by the summation of all five heavy metals, and the weight of parameter (Wp) was calculated as per Eq. (2). Table S4 presents the Wp for each heavy metal analysed in this study. Status of contamination (Sc) was calculated by dividing measured concentration as per Eq. (3). S_c_ for each sample point analysed in this study is given in table S5. HPI (Heavy metal Pollution Index) was estimated by multiplying concentration status with the parameter weight as per Eq. (4).

1 $$Rw =\frac{1}{{Permissible}_{limits}}$$

2 $$Wp =\frac{{R}_{w}}{\sum {R}_{w}}$$

3 $$Sc =\frac{{Measured}_{conc.}}{{Permissible}_{limits}}$$

4 $${\text{HPI }} = {\text{ Wp }} \times {\text{Sc}}$$

5 $${\text{HPI}}_{{{\text{total}}}} = {\text{ HPI}}_{{({\text{Cd}})}} + {\text{ HPI}}_{{({\text{Cu}})}} + {\text{ HPI}}_{{({\text{Fe}})}} + {\text{ HPI}}_{{({\text{Pb}})}} + {\text{ HPI}}_{{({\text{Zn}})}}$$

### Health risk assessment

Heavy metals pose health risks upon ingestion. Hence it calls for health risk assessment. Several studies have adopted health risk assessment^4^. The proposed model helps determine possible health risks from exposure to pollutants heavy metal^8^. This study also used the same model for estimating health risk posed from heavy metals. Health risk is categorized as cancer causing (carcinogenic risk) and risk beside cancer (non-carcinogenic risk)^5^. Health risk assessment requires an average daily intake (A.D.I.) as per Eq. (6). ADI for each heavy metal in the study area is presented in Table S7. Which further needs the body weight (70 kg), measured heavy metal concentration (C), daily intake of water (I.R., 2 L day^−1^), Exposure frequency (E.F., 365 days), exposure duration (E.D., 70 years) and Average time (AT, exposure duration x exposure frequency). It has to be noted that several studies have changed the values of these parameters as per local conditions. Exposure duration has been reported to be taken as 30 years, 65 years etc*.*^7,34^. Also, exposure duration for every region will vary depending on life expectancy. Park et al.^7^ has used a daily intake value of 1.5 L day^−1^. After A.D.I. is estimated, the non-carcinogenic risk is calculated by dividing A.D.I. with reference dose (*RfD*) of respective heavy metal in concern to determine any possible adverse health effect as per Eq. (7) in the form of Hazard quotient (H.Q.). However, when several parameters are concerned, they are all summed up to calculate the hazard index (H.I.) per Eq. (8). Similarly, carcinogenic risk (C.R.) is obtained by multiplying A.D.I. by cancer slope factor (C.S.) as per Eq. (9) and summation of all C.R. gives carcinogenic risk index (CI) as per Eq. (10).

6 $$\mathrm{ADI }=\frac{{C \times IR \times EF \times ED}}{{BW \times AT}}$$

7 $$\mathrm{HQ }=\frac{ADI}{RfD}$$

8 $${\text{HI }} = {\text{ HQ}}_{{({\text{Cd}})}} + {\text{ HQ}}_{{({\text{Cu}})}} + {\text{ HQ}}_{{({\text{Fe}})}} + {\text{ HQ}}_{{({\text{Pb}})}} + {\text{ HQ}}_{{({\text{Zn}})}}$$

9 $${\text{CR }} = {\text{ ADI }} \times {\text{ CS}}$$

10 $${\text{CI }} = {\text{ CR}}_{{({\text{Cd}})}} + {\text{ CR}}_{{({\text{Cu}})}} + {\text{ CR}}_{{({\text{Fe}})}} + {\text{ CR}}_{{({\text{Pb}})}} + {\text{ CR}}_{{({\text{Zn}})}}$$

## Machine learning models

This study employed artificial neural network, support vector regression and random forest regression approaches for forecasting.

### Artificial neural network

The ease of use makes artificial neural network (ANN) mostly used machine learning model in groundwater modelling^19^. ANN typically architecture consists of three layers which comprise of a output layer followed by hidden layer and input layer. The neurons that exhibit weight and bias are what link these layers together. The variables are changed from the layer tth to the layer t^+^ 1st by an activation function (*f*) and continue in this manner until they reach the target layer. weights and biases are changed /altered of the layers, and model is trained by repeated iteration till satisfactory results are achieved. We use models with three MLP layers to make this strategy more straightforward, and the outputs (X_k_) are estimated using the following equations:

11 $${\mathrm{X}}_{k} = {\mathrm{y}}_{k}\left( \sum_{i=1}^{m}{A}_{tk}* {f}_{t} \left(\sum_{i=1}^{n}{X}_{i}{W}_{it}\right)\right)+ {\mathrm{A}}_{0}$$

With A_0_ being the bias, n being the feature numbers, neuron number in hidden layer is presented as m, and the number of neurons in target layer is denoted as p. the weight in between tth and kth target neuron is represented by A_tk_. While, the weight between ith and tth neuron is given as A_it_. The transfer function in output layer k is presented as y_k_. the transfer in hidden layer t is denoted as f_t._

### Random forest

Collection of decision tree-based machine learning models, forms the basic architecture of random forest (RF) approach. Regression and classification are carried out by combining a method that generate a model which assembles optimized decision trees. The decision trees are identified by varying/switching/altering the covariates during training process and is selected based on best performance^35^. The assigned weights to each tree output the goal is met or not is determined. Several variables and trained trees are a vital requirement to run RF model successfully. These parameters are crucial to the model's stability and, consequently, to the accuracy of predictions^19^. In this work, we use the trial-and-error method to pick the model parameters.

### SVM algorithm (support vector machine)

It is highly used in research work attributed to its ability to perform regression and classification functions. This is achieved by developing hyper plane which results in reduction of errors thereby increasing the efficiency and performance of the model. The modelling system is denoted as S and Ds represents the observation dataset. Ds = (xi, yi)n, i = 1, where xi and yi are represented by linear functions that reflect the inputs and outputs, respectively, as illustrated in Eq. (12).

12 $${\text{f}}\left( {\text{x}} \right) \, = (\alpha \, * \, \theta \, \left( {\text{x}} \right) \, + {\text{ b}})$$

The minimizing of the function is as per Eq. (13) as per Eq. (14) which is the ideal function. Therefore, loss functions such the quadratic, Hubber, and -insensitive approaches can be used.

13 $$\mathrm{min }\left(\mathrm{\alpha },\mathrm{ b},\upupsilon -,\upupsilon +\right)={\frac{1}{2}}^{ *} \Vert {\alpha }^{2}\Vert +\sum_{i=1}^{n}\left({\alpha i}^{-}- {\alpha i}^{+}\right)$$

14 $$\mathrm{S}.\mathrm{t}\left\{\begin{array}{c}{y}_{i}- {\alpha }^{T}* \theta \left(x\right)-b \le \upepsilon + \upsilon {\mathrm{ i}}^{-} \\ -{y}_{i}+ {\alpha }^{T}* \theta \left(x\right)+b \le \upepsilon + \upsilon {\mathrm{i}}^{+}\\ {vi}^{-}, {vi}^{+} \ge 0\\ i=\mathrm{1,2}\dots n\end{array}\right\}$$

Linear functions, and b stand for the basis and weight vectors, respectively, and C is θ(x) a Kernel function (k) like a polynomial or radial basis set at a predetermined value to remove training error, and lower and higher output constraints of i and i respectively. The Radial Basis Function (RBF) is estimated using Eq. (15).

15 $$k(\mathrm{xi},\mathrm{ xj}) ={exp}^{(-y|{x}_{i}- {\left.{x}_{j}\right|})}\mathrm{exp}(-y|{x}_{i}- {\left.{x}_{j}\right|})$$

### Modelling generalization and validation

It is important to note the sources on which the data were based, including the field and lab data gathered for this study. Based on the in-situ measurements from the field survey, groundwater samples were collected and analysed in the lab. The generated model was evaluated based on statistical analysis derived from mean absolute error (MAE), root means square error (RMSE) and mean absolute percentage error (MAPE). Prior to modelling, k-fold cross-validation was used as external validation to improve the output, lower error uncertainties, and boost integrity.

16 $$\mathrm{RMSE }=\sqrt{\frac{\sum_{i=1}^{N}({a}_{i}-{a}_{e}{)}^{2}}{N}}$$

where, RMSE = root mean square error, i = variable, a_i_ = actual readings, a_e_ = predicted values

17 $$\mathrm{MAE }=\frac{\sum_{i=1}^{n}\left|{a}_{e}- {a}_{i}\right|}{n}$$

MAE = mean absolute error, a_e_ = predicted values, a_i_ = actual values, n = data points (number)

18 $$\mathrm{MAPE }=\frac{1}{n} \sum_{t=1}^{n}\left|\frac{{a}_{i}- {a}_{e}}{{a}_{e}}\right|$$

MAPE = mean absolute percentage error, N = number of summation iteration occurred, a_e_ = predicted value, a_i_ actual value.

### Modelling results and discussion

Prior to training, data was saved in .csv file. The data was divided into 70–30% for training and validation purpose. The Machine learning algorithms were run using MATLAB program. Trial and error approach was adopted to identify functions and parameters for each model used in this study. The optimal parameters and functions used in this study are used given in Table 1. The assumptions of the three algorithms are given in Table S2.

**Table 1:** Functions and parameters used during training phase.

Model	Parameters and functions
Random forest	Number of trees = 15
SVM	e = 0.015, function loss RBF γ = 1.2, kernel function C = 200
ANN	Layers = 3 Hidden layer neurons = 9 Algorithm = Levenberg–Marquardt Activation function = sigmoid Epoch number = 1000 Learning rate = 0.01

**Figure 2:**
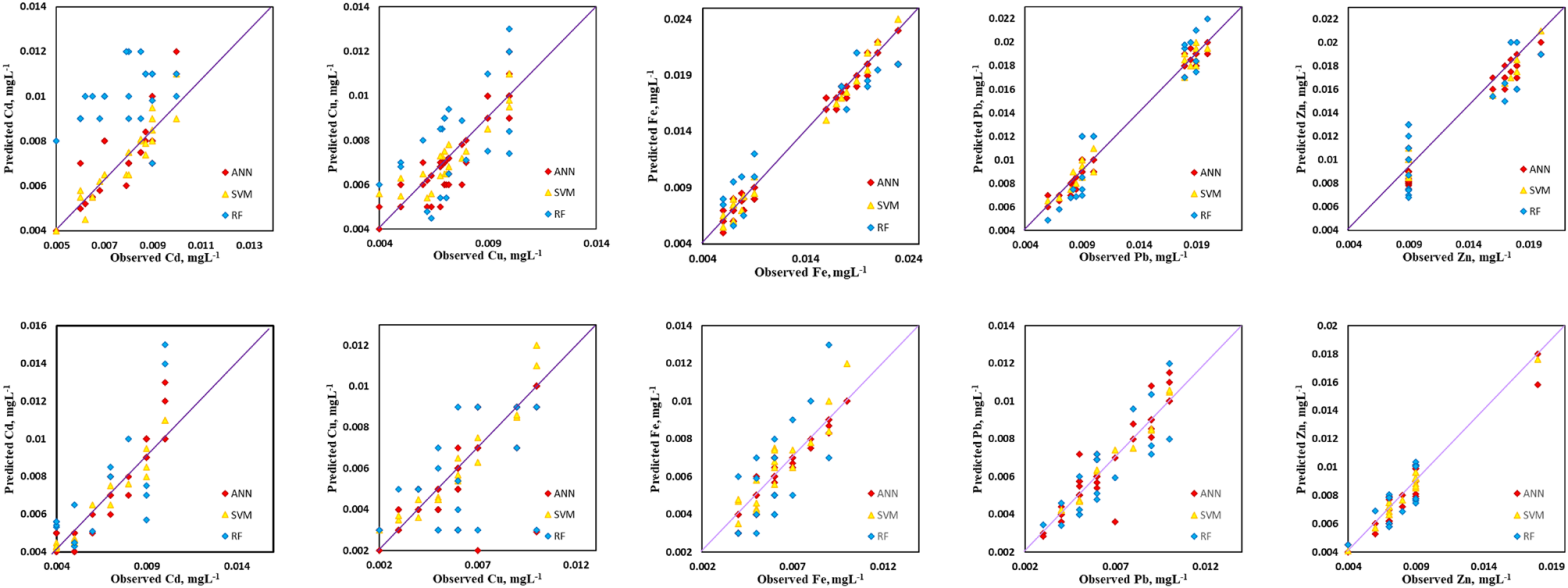
Scattered plot of heavy metals Cd, Cu, Fe, Pb and Zn of the observed and simulated values top row (winter) and bottom row (spring).

Based on the model performance as presented in Table 2. It is evident that ANN model has higher performance. The SVM model follows closely to ANN model and SVM model is not close to other two models. Figure 2 shows the scattered plot of predicted and observed heavy metal concentration in the rivers of Mohammedia prefecture. When the values are distributed evenly on both sides are termed as satisfactory as per Gaussian distribution^19^. Additionally, the Random Forest model, with the exception of the MAR parameter, predicts values that are fairly widely distributed throughout the X^=^Y line, while the ANN shown greater performance when the projected values are much closer to the observed values. The SVM and ANN models, for heavy metals Cd, Cu, Fe, Zn, and Pb, respectively, revealed rather excellent value distributions over the XY line. Importantly, even though it performed reasonably well during the training phase, the RF models failed to replicate the parameter since the projected values are off from the Y^=^X line and have RMSEs of more than 10%. These findings show that the ANN and SVM are more accurate at predicting the analyzed irrigation water quality metrics than the RF model.

**Table 2:** Validation phase model performance.

Parameter	RF	SVM	ANN
Cd^2+^			
MAE	0.00031	0.00102	0.0038
MAPE	2.05	4.52	9.8
RMSE	0.0006	0.0013	0.00592
Cu			
MAE	0.0007	0.00138	0.00401
MAPE	4.07	5.29	28.75
RMSE	0.00107	0.002	0.00611
Fe			
MAE	0.00091	0.00142	0.00476
MAPE	7.1	9.8	32.54
RMSE	0.00169	0.00233	0.00775
Pb			
MAE	0.00071	0.0021	0.00345
MAPE	1.98	5.24	23.2
RMSE	0.0005	0.0025	0.00732
Zn			
MAE	0.00025	0.00121	0.00412
MAPE	2.1	6.78	11.23
RMSE	0.00102	0.0023	0.00654

Based on the Table 2 the performances of the models used in this study can be presented as follows: ANN performed better in prediction of all heavy metals as compared to SVM model followed by RF model. Except of RF the other two models were observed to be unbiased. Table S1 gives the values of R^2^ for the three models used in this study. Based on R^2^ values of the three models ANN was most fitting. For Zn during spring season all three models gave optimum fitting R^2^ value in range of 0.98–0.99. Cd value of R^2^ for spring season was satisfactory with range 0.89–0.96 for SVM and ANN models. Similar values were observed for Pb during spring season with R^2^ values in range of 0.999–0.998 for ANN and SVM models respectively. For Cu and Fe, the RF and SVM models did not fit satisfactorily, the only best fitting model was ANN with R^2^ value of 0.99. During winter season the best fitting model for Zn prediction were observed to be RF and ANN with R^2^ values of 0.96 and 0.99 respectively. the fitting model for other heavy metals during winter season was found to be only ANN with R^2^ range of 0.82–0.99 for Cd, Cu, Fe, and Pb. The other two models SVM and RF did not even come close to ANN model in terms of R^2^ values.

### Geospatial distribution and prediction

**Figure 3:**
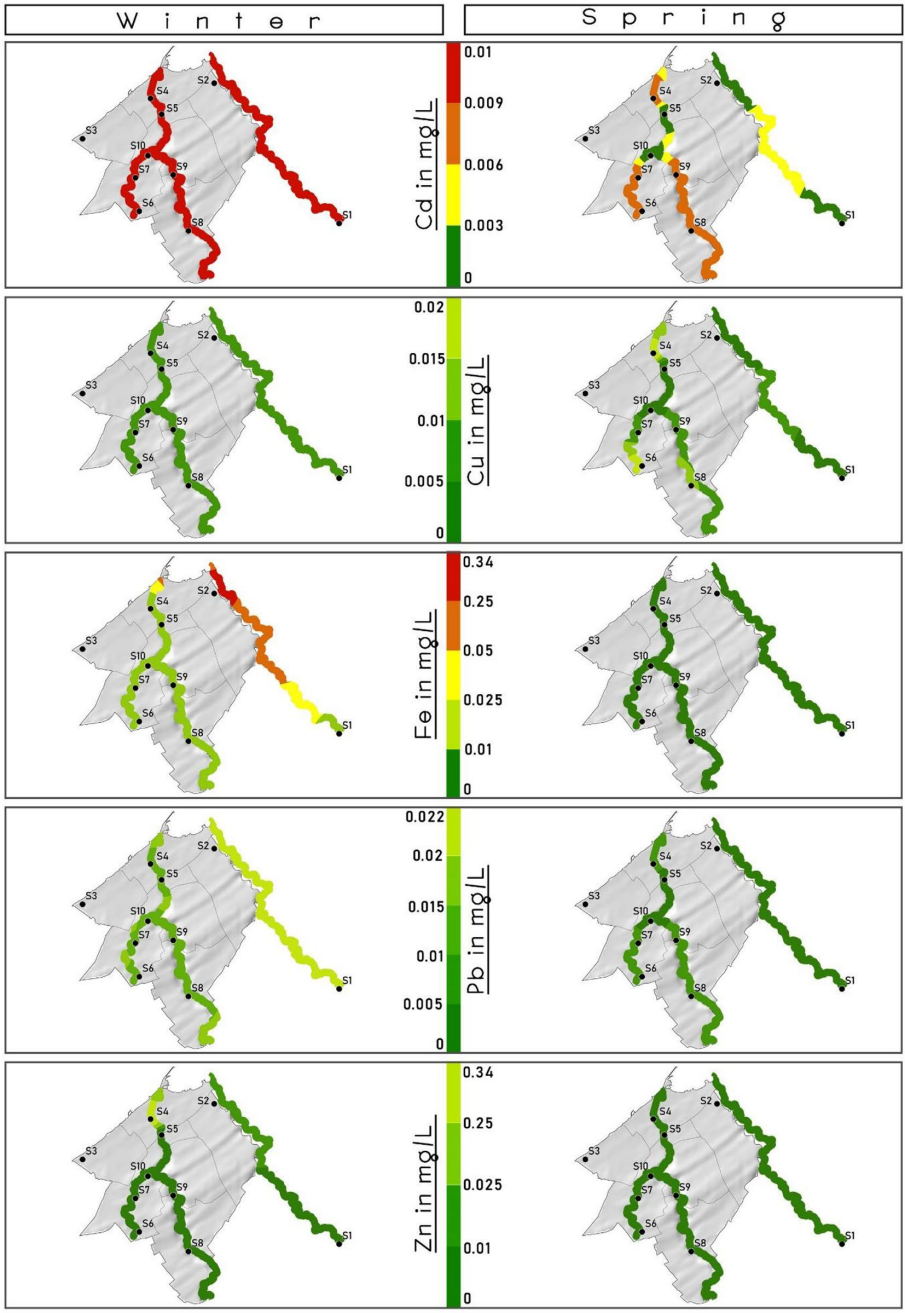
Spatial distribution of heavy metal in the three rivers of Mohammedia prefecture during winter and spring season.

**Figure 4:**
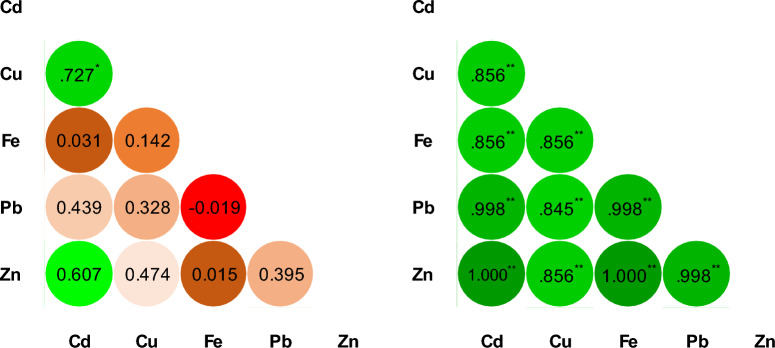
Correlation matrix of heavy metal during winter (left) and spring (right) season.

**Figure 5:**
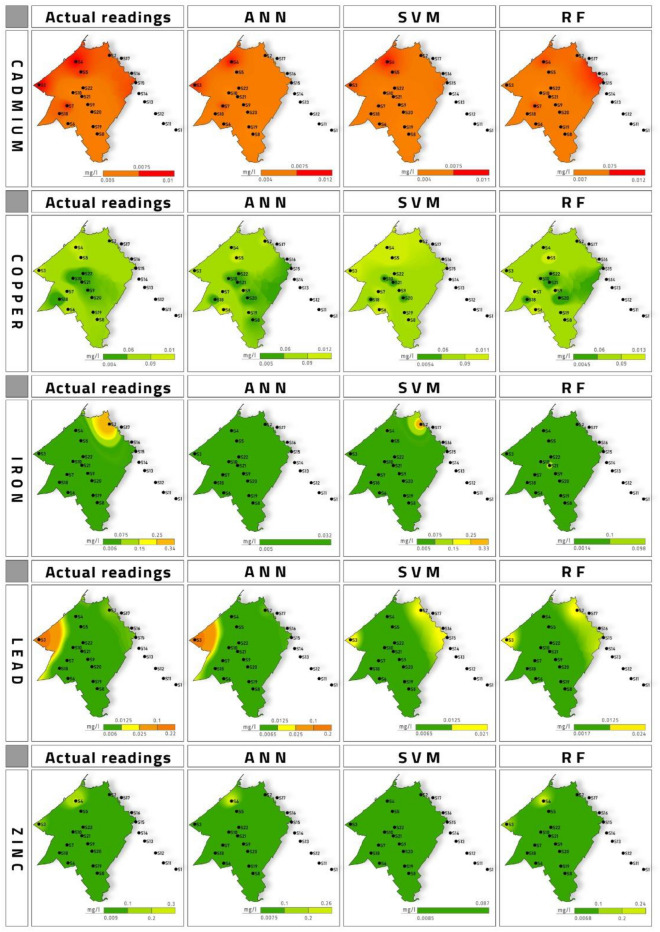
Comparison of geospatial distribution of actual and predicted readings from SVM, RF and ANN models for winter season.

**Figure 6:**
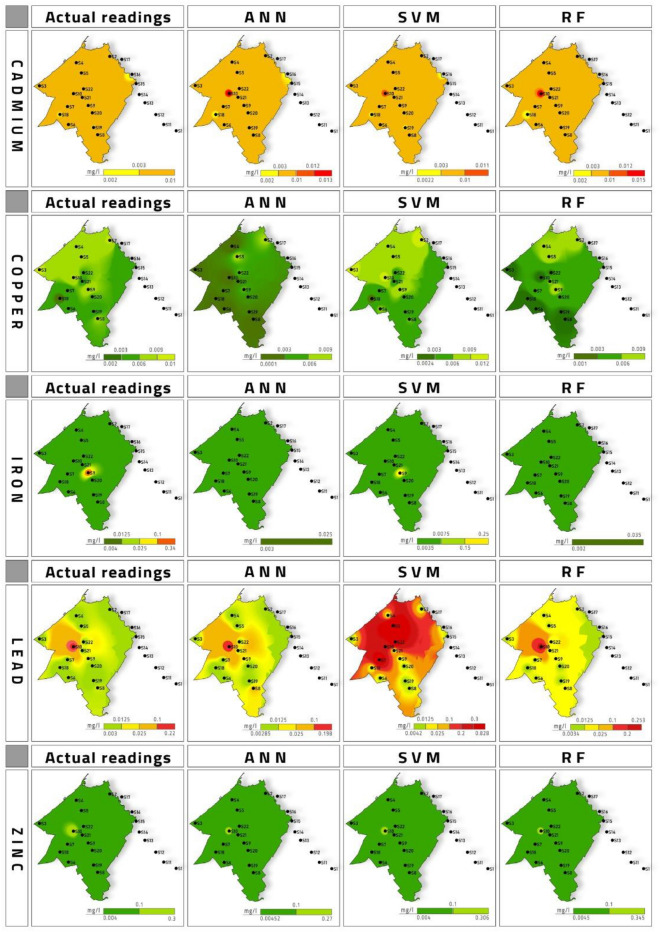
Comparison of geospatial distribution of actual and predicted readings from SVM, RF and ANN models for spring season.

Geospatial distribution of heavy metals along the length of the target rivers is presented in Fig. 3. The correlation matrix of the heavy metals present in the three rivers is presented in Fig. 4. The occurrence of heavy metal during the winter season was in order Cd and Cu > Zn > Fe > Pb. This order changed to Cu > Pb > Zn > Cd and Fe in winter. The predicted values from the models were used to develop geospatial maps for Mohammedia prefecture for better understanding as just along the length and width of the river the visualization of performance accuracy of the models is greatly restricted. The generated maps are presented in Figs. 5 and 6 for comparison.

Cadmium was initially used as a substitute for tin. But later on, it was widely used as a pigment in the paint industry. Another major source is its utilization as an electrode in alkaline batteries^36^. Cd in higher concentrations poses a threat to the kidney, vascular system and kidney^37^. Prolonged Cd exposure has also been responsible for Osteopenia in the human body^38^. Cd interferes with Ca metabolism, thereby comprises bone health. Also, it inhibits vitamin D absorption, impacting the renal function's performance. Plating operators welding workers are examples of occupational exposure to Cd, which are highly susceptible to Osteopenia and osteoporosis besides ingestion of Cd through drinking water^38^. The geospatial distribution of the predicted Cd values indicated that ANN model was the most accurate followed by SVM model. RF model values were far from the actual Cd values during winter season. While during Spring season, all the models’ geospatial maps were showing high values are sample 10 which rendered them with low accuracy. Based on prediction value with variation at sample 16 it was observed that ANN and SVM accurate. RF model value-based maps were most accurate.

Copper has a daily oral intake limit of 1–2 mg per capita. Cu is an essential trace element. Corrosion of copper pipes is considered the primary source of Cu in the drinking water supply^39^. Cu act as a catalyst for many redox enzymes, iron metabolism, antioxidant defence and immune function^40^. Low pH with high Cu concentration can lead to the death of exposed fish^41^. Cu acute toxicity can cause nausea^39^. High concentrations lead to oxidative damage to the cell and its death from Fenton-type redox reactions^40^. The Cu prediction values were used to generate geospatial maps which showed that ANN and RF models were more accurate. SVM model was the least accurate model for Cu values during winter season. During spring the accuracy for Cu prediction was most accurate by SVM model followed by RF while ANN was the least accurate model.

From 4 million tons of Pb being consumed, only 1 million tons are recycled globally. The remaining 3 million tons of Pb is dumped into the environment contaminating Air, Soil and Water^38^. Carpet toys and paints in the home are the primary sources of Pb exposure to children^42^. Pb is a nonessential toxic heavy metal that poses a high risk to human health. Pb is identified as primarily responsible for heavy metal poisoning in children^43^. Chronic exposure to Pb can lead to brain damage, kidney damage, congenital disability, autism, psychosis, paralysis and may cause death^44^. The Fe prediction during winter season was plotted as geospatial maps. The most accurate model was observed to be SVM. The ANN and RF model based geospatial maps were observed to be nowhere near to the maps generated based on actual Fe values. During spring season also SVM was observed to be the most accurate model.

Despite being together with two toxic metals, mercury and cadmium, Zn is considered a relatively non-toxic metal in the periodic table^45^. Zn is absorbed by small intestines upon ingestion and is found in all body fluids and tissues^46^. Zn occurrence in groundwater can be attributed to infiltration from fertilizers used in agriculture^47^. Pb geospatial maps during winter season is presented in Fig. 5. The most accurate map was observed to be ANN model based predicted values. SVM and RF model based generated geospatial maps it can be inferred that they were not accurate as compared to ANN. For spring season most accurate model was observed to be ANN followed by RF model and least accurate model was the SVM.

A sample points 8 and 9 in El Maleh river, and detection frequency was 100% for Cd, Fe, Cu, Zn and Pb in the winter and spring. This indicated that the main river of Mohammedia prefecture El Maleh was already laden with heavy metal before entering Mohammedia prefecture. At point P8 and P9, Cd (0.006–0.009 mg L^−1^), Cu (0.006–0.007 mg L^−1^), Fe (0.006–0.009 mg L^−1^), Pb (0.008–0.009) and Zn (0.009 mg L^−1^) was observed which increased to Cd (0.01 mg L^−1^), Cu (0.01 mg L^−1^), Fe (0.009 mg L^−1^), Pb (0.01 mg L^−1^) and Zn (0.009–0.3 mg L^−1^) at the point S4 and S5 crossing Mohammedia city. This increase in heavy metal concentration is attributed to wastewater effluent from Mohammedia city. However, the concentration for all five heavy metals in the spring season was almost the same, indicating that the river was already polluted before entering the Mohammedia prefecture. Cd was the only heavy metal above the acceptable limit of 0.003 mg L^−1^ in the El Maleh river in every water sample. Cu, Fe, Pb, Zn were well below permissible limits. Geospatial map of Zn for the actual and prediction model for winter season shows that ANN RF were the most accurate model. The SVM model prediction map shows it was not fit to be considered for prediction of Zn. During spring season based on geospatial map all the models can be said to be accurate for prediction.

Being the tributary, Hassar river results followed a similar trend in terms of seasonal variation. During winter season, at point S4 and S5, Cd (0.007–0.009 mg L^−1^), Cu (0.01–0.009 mg L^−1^), Fe (0.007 mg L^−1^), Pb (0.009 mg L^−1^), Zn (0.009) mg L^−1^. In the Spring season, Cd^2+^, Cu Zn, Pb, and Fe increased to 0.009 mg L^−1^ concentration. At point S10, the merging point of river Hassar and El Maleh, all the five heavy metals were below detection level in the spring season. However, based on winter season it can be observed that there was dilution effect on heavy metal concentration with Cd^2+^ 0.005 mg L^−1^, Cu 0.005 mg L^−1^, Fe 0.006 mg L^−1^, Pb 0.006 mg L^−1^, and Zn 0.009 mg L^−1^. Following the trend of El Maleh river water, Cd^2+^ was the only heavy metal found to be above the permissible limits.

Nfifikh river is a border river of Mohammedia prefecture. Point S1 was taken outside the prefecture to assess the potential increase in heavy metal concentration while crossing Mohammedia prefecture. Cd^2+^ concentration decreased to 0.008 mg L^−1^ from 0.009 mg L^−1^. However, Fe, Cu, Pb and Zn increased to 0.009 mg L^−1^, 0.34 mg L^−1^, 0.02 mg L^−1^, and 0.06 mg L^−1^ from 0.007 mg L^−1^, 0.018 mg L^−1^, 0.019 mg L^−1^ and 0.018 mg L^−1^ respectively. In the spring season, the heavy metal concentration was below detection level at S1 and S2. Edokpayi et al.^48^ has suggested high infiltration as a factor for increased heavy metal concentration in groundwater. However, in this study, the infiltration rate has to be higher during the winter season as it receives an average of 30 mm of precipitation. During the spring season, this precipitation reduces to 16 mm. Hence this possibility has to be ruled out in the present scenario.

The coastal lagoon point S3 was evaluated as it was also a surface water body named Douar eel Marja in Mohammedia prefecture. In winters the heavy metal concentration was; Cd^2+^ 0.01 mg L^−1^, Cu 0.01 mg L^−1^, Fe 0.02 mg L^−1^, Pb 0.22 mg L^−1^ and Zn 0.17 mg L^−1^. The heavy metal concentration in spring was contrary to the three rivers investigated in this study. Cd^2+^ was above the permissible limit but was reduced to 0.009 mg L^−1^, Cu 0.01 mg L^−1^, Fe 0.009 mg L^−1^, Pb 0.009 mg L^−1^ and Zn 0.009 mg L^−1^. Ruiz et al.^16^ has investigated Nador lagoon and reported concentration of Cu (46–96 mg/kg), Zn (140–212 mg/kg), and Pb (41–73 mg/kg). Nador lagoon heavy metal concentration has been attributed to effluent discharge from wastewater treatment plants and runoff from old iron ore mining areas. Ait Alla et al.^49^ has also attributed wastewater discharge to causing a change in water quality of river Souss estuary in the bay of Agadir in Morocco to the degree which will affect biodiversity. However, sample point S3, despite being located in the industrial zone, heavy metal concentration is not as high as reported in other studies, which can be attributed to only one fact that water entering the lagoon was treated before.

During the winter, the P-value < 0.05 for Cd^2+^, Cu, and Zn indicated their same-origin source. Which again was validated by correlation values of 0.727 and 0.607 between them. However, there was a strong correlation between heavy metals in the spring season. Cd^2+^, Fe, and Zn had a correlation value of 1. Pb, and Cd, had a correlation value of 0.998. Cd^2+^, Cu, Pb and Zn had a correlation value of 0.85. Also, the p-value < 0.01 for Cd^2+^, Fe, Cu, Pb and Zn presented a very strong relationship. The correlation matrix is presented in Fig. 3. This indicates that the river's heavy metals were of the same origin. The concentration of heavy metal except for Cd^2+^ was under permissible limits. However, this does not relieve the risk from heavy metal contamination and requires risk assessment in terms of pollution and human health^50^. Even though some heavy metals are essential for the body biological mechanism and metabolism, they can also have a toxic effect on the human body at higher doses^51^.

The geospatial distribution comparison between simulated values and observed values for ANN, SVM and RF approach is presented in Figs. 5 and 6 for winter and spring season respectively. Geodistribution map was observed.

### Heavy metal pollution index and health risk assessment

**Figure 7:**
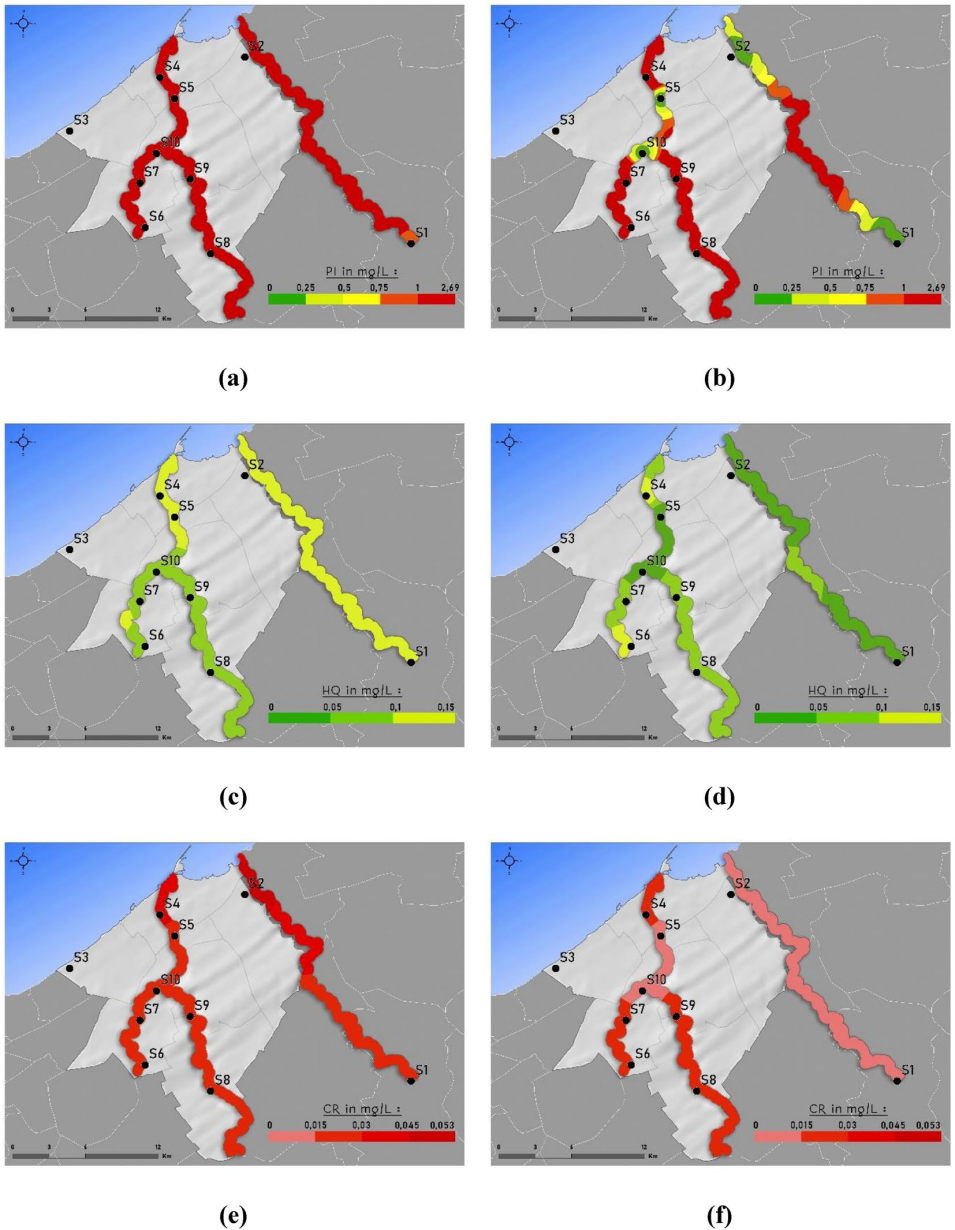
Spatial distribution of Pollution index (**a**) and (**b**), Hazard quotient (**c**) and (**d**) and carcinogenic risk (**e**) and (**f**) in Nfifikh, Hassar and El Maleh river during winter (left) and spring (right).

The indexing approach is commonly used to assess water quality in several types of research^5,52,53^. Sarti et al.^54^ Have employed an indexing approach to assessing groundwater quality in Morocco. The indexing approach helps in the degerming degree of pollution and the water body^55^. Heavy metal pollution index is similar to the Water quality index (W.Q.I.) approach but incorporates the weightage factor against each weight factor of heavy metal which improvisation over W.Q.I. approach^33,56^. HPI index value 0–0.25 signifies negligible pollution (Excellent water), 0.25–0.5 means very low pollution (Good water), 0.5–0.75 denotes low pollution (poor water), 0.75–1 depicts moderate pollution (very poor water), values > 1 means high pollution (not fit for drinking)^57^. The difference in W.Q.I. and H.P.I. is based on scale, i.e., W.Q.I. is presented on a scale of 1–100 while H.P.I. in this study is presented on a scale of 0–1. Based on the H.P.I. values in this study, the waters of rivers Nfifikh, Hassar and El Maleh are highly polluted. The H.P.I. index value ranged was in between 2 and 3. Hossen et al.^58^ has proposed another heavy metal pollution index scale where H.P.I. 1–3 is deemed moderately polluted. However, it has to be noted that Hossen et al.^58^ was scaling pollution level. While in this study, we were determining the water quality in terms of drinking. Nevertheless, Cd was found to be the major contributor to the pollution index to such an extent if Cd is taken out, the water quality will reduce to excellent. The spatial distribution of H.P.I. in Mohammedia prefecture during the winter and spring seasons is presented in Fig. 7a and b.

Figure 7c and d presents the non-carcinogenic risk from heavy metals presence in the river water. Non-carcinogenic risk assessment is conducted to determine the probability of adverse health effects on human health^55^. In this study, heavy metal contamination from heavy metal was considered for non-carcinogenic risk assessment. Non-carcinogenic risk is presented as hazard quotient (H.Q.) or risk quotient (R.Q.). H.Q. value < 1 signifies that there is no carcinogenic risk^24^. H.Q. > 1 indicate a potential health risk^5^. In the case of multi parameters, H.I. is used, which indicates summed up H.Q. values for each heavy metal. H.I. value for winter was in the range of 0.08–0.52. at the same time, H.I. for spring was in the range of 0.09–0.12. The spatial distribution of H.I. in rivers of Mohammedia prefecture is presented in Fig. 5. River Nfifikh had H.I. values between 0.11 and 0.15, Hassar 0.08–0.09 and El Maleh 0.05–0.13. Based on the result of the health risk assessment in the present scenario, the water of the three rivers does not pose an adverse health risk.

The spatial distribution of C.R. in the rivers of Mohammedia prefecture is presented in Fig. 7e and f. Carcinogenic risk (C.R.) assessment is carried out only for pollutants with carcinogenic properties^24^. Hence heavy metal contamination necessitates its assessment. C.R. risk assessment is not on a scale similar to hazard quotient, but it is probability ratio of acquiring cancer and is defined by the ratio of 1:1,000,000 to 1:10,000^31^. This indicates that it is acceptable if 1 in every 1,000,000 and up to 10,000 can get cancer upon exposure to the carcinogenic pollutant. Nfifikh, Hassar, and El Maleh river CI index indicated that 1 in every 100 persons has the probability of getting cancer upon consumption of water over a lifespan of 70 years in both seasons. Nfifikh Cd and Fe were mainly responsible for carcinogenic risk in the river. However, in river Nfifikh CR risk is not attributed to pollutant discharge from Mohammedia prefecture as C.R. value of point S1 was 0.025, but in Mohammedia prefecture S2, the value was reduced to 0.022. Hassar and El Maleh Cd were solely responsible for carcinogenic risk in the river. Also, it was observed that CI risk values reduced at final sample points of El Maleh river to 0.017 and 0.021 at S8 and S9, respectively, as compared to CI 0.032 and 0.026 at sample points S4 and S5, respectively. This validates that Mohammedia prefecture was not responsible for C.R. risk from eh waters of the three rivers, and the previous urban and industrial areas need to be investigated for further confirmation.

## Conclusion

This study was conducted to analyse the occurrence of heavy metal, its seasonal variation and potential health risk assessment in the three rivers of Nfifikh, Hassar and El Maleh in Mohammedia Prefecture, Morocco. This was first of its kind of study in Mohammedia prefecture to evaluate surface water bodies in terms of heavy metal contamination. The study was also first to forecast heavy metal occurrence in surface water bodies using artificial and machine learning algorithms in Mohammedia prefecture of Morocco.

All five heavy metals Cd, Cu, Pb, Zn and Fe were detected with 100% frequency at all sampling locations. Iron, lead, copper, and zinc were below permissible limits in all sample locations in the three rivers. The heavy metal concentration in river water samples were in range of 0.005–0.01 mg L^−1^, 0.005–0.01 mg L^−1^, 0.007–0.34 mg L^−1^, 0.007–0.22 mg L^−1^ and 0.009–0.3 mg L^−1^ for Cd, Cu, Fe, Pb and Zn respectively. Cadmium was the only heavy metal found above the permissible limits in all three rivers. Heavy metal pollution index indicated cadmium was solely responsible for water quality deterioration with index values ranging between 1 and 2 both in winter and spring season. The heavy metal pollution index indicated the water in the high pollution category, which rendered it unfit for direct human consumption.

Upon non-carcinogenic risk assessment, the HQ values were observed to be less than 1 in both seasons and in all three rivers. The hazard quotient revealed no potential adverse health impact on consumption. However, the carcinogenic risk was in lieu with heavy metal pollution index and revealed the carcinogenic risk is high upon consumption of river water without treatment. The carcinogenic risk assessment revealed that 1 in every 100 person is susceptible to cancer risk. Cadmium was again the main pollutant responsible for the cancer risk. The other heavy metals iron, zinc, lead and copper posed cancer risk in range of lower acceptable limit of 1in 10,000 person. Also, it was observed that Mohammedia prefecture was not contributing to the heavy metal occurrence in the three rivers as it was already laden with heavy metal before it entered the lands of the prefecture.

Artificial neural network and support vector machine approach are most suitable for prediction. Random forest approach was not found to be accurate enough to be considered suitable for prediction. The geospatial maps gave a new insight in prediction efficiency as it does not solely rely on statistical approach. It serves as a more comprehensive tool for decision and policy makers. The geospatial maps in this study are limited to study area digitization. Higher accurate maps can be obtained if they are generated based on the aquifers present in the prefecture. This will present the actual and real-time scenario and will be highly effective in managing water resources sustainably.

Cadmium was solely responsible for water pollution and carcinogenic risk in the river's waters in Mohammedia prefecture. Hence this study narrowed down the major task of planners and decision-makers by identifying the main pollutant. This will enable the governing agencies to adopt measures to mitigate occurrence and pollution from cadmium in river water. Further studies are required to identify the sources of pollution in river water before it enters Mohammedia prefecture. Also, the health data of the resident population needs to be investigated to determine the actual health impact on the population. This study analyzed only five heavy metals. Future studies can incorporate arsenic and other heavy metals to identify other major pollutants such as cadmium to ensure optimized and sustainable surface water sources in Mohammedia prefecture specifically and Morocco in general.

### Supplementary Information

Supplementary Tables.

## Data Availability

The data that support the findings of this study are available from [Roohul Abad Khan]. Still, restrictions apply to the availability of these data, which were used under license for the current study, and so are not publicly available. However, data are available from the authors upon reasonable request and with permission of [Roohul Abad Khan].
