# Supplementary material for: Geospatial distribution and machine learning algorithms for assessing water quality in surface water bodies of Morocco
Source: Sci Rep. 2023 Nov 23;13:20599. doi: 10.1038/s41598-023-47991-z (PMC10667218; doi:10.1038/s41598-023-47991-z)
Supplement: Supplementary file 1 — Supplementary Tables. [file 41598_2023_47991_MOESM1_ESM.docx]

**SUPPLEMENTARY FILES**

**Table S1** R^2^ Values for each modelled heavy metal

| **SPRING** | | | |
| --- | --- | --- | --- |
|  | **RF** | **SVM** | **ANN** |
| **Cd** | 0.6542 | 0.9614 | 0.8994 |
| **Cu** | 0.0054 | 0.0243 | 0.9232 |
| **Fe** | 0.0014 | 0.4775 | 0.999 |
| **Pb** | 0.005 | 0.9982 | 0.999 |
| **Zn** | 0.9804 | 0.9991 | 0.9971 |
| **WINTER** | | | |
|  | **RF** | **SVM** | **ANN** |
| **Cd** | 0.14 | 0.6798 | 0.8757 |
| **Cu** | 0.3551 | 0.6881 | 0.8211 |
| **Fe** | 0.0064 | 0.4251 | 0.9569 |
| **Pb** | 0.2058 | 0.1813 | 0.9995 |
| **Zn** | 0.9637 | 0.0004 | 1 |

**Table S2 Assumptions of the algorithms**

| **SVM** | **ANN** | **GA** |
| --- | --- | --- |
| Data is linearly separable | Artificial Neurons are arranged sequentially | Fitness function quantifies the potential of solutions |
| Clear distinction between different data classes | There is no interaction between neurons of each layer | Mapping of bit strings to generate potential solutions |
| Data is normalized in SVM | Same level hidden layers have same activation function |  |
| The input data is on same scale without dominance of any particular parameter | Each interconnected neural network is associated with its respective weight |  |
| Data is homogenously distributed in training and testing phase |  |  |

**Table S3** Relative weight of the heavy metals

|  | **Rw** | | | | |
| --- | --- | --- | --- | --- | --- |
| **Sample points** | **Cd** | **Cu** | **Fe** | **Pb** | **Zn** |
| S1 | 333.3333 | 0.5 | 4 | 40 | 0.333333 |
| S2 | 333.3333 | 0.5 | 4 | 40 | 0.333333 |
| S3 | 333.3333 | 0.5 | 4 | 40 | 0.333333 |
| S4 | 333.3333 | 0.5 | 4 | 40 | 0.333333 |
| S5 | 333.3333 | 0.5 | 4 | 40 | 0.333333 |
| S6 | 333.3333 | 0.5 | 4 | 40 | 0.333333 |
| S7 | 333.3333 | 0.5 | 4 | 40 | 0.333333 |
| S8 | 333.3333 | 0.5 | 4 | 40 | 0.333333 |
| S9 | 333.3333 | 0.5 | 4 | 40 | 0.333333 |
| S10 | 333.3333 | 0.5 | 4 | 40 | 0.333333 |

**Table S4** W_p_ values obtained by dividing each pollutant weight by summation of all weights of pollutants

|  | Wp | | | | |
| --- | --- | --- | --- | --- | --- |
| **Sample points** | Cd | Cu | Fe | Pb | Zn |
| S1 | 0.881445 | 0.001322 | 0.010577 | 0.105773 | 0.000881 |
| S2 | 0.881445 | 0.001322 | 0.010577 | 0.105773 | 0.000881 |
| S3 | 0.881445 | 0.001322 | 0.010577 | 0.105773 | 0.000881 |
| S4 | 0.881445 | 0.001322 | 0.010577 | 0.105773 | 0.000881 |
| S5 | 0.881445 | 0.001322 | 0.010577 | 0.105773 | 0.000881 |
| S6 | 0.881445 | 0.001322 | 0.010577 | 0.105773 | 0.000881 |
| S7 | 0.881445 | 0.001322 | 0.010577 | 0.105773 | 0.000881 |
| S8 | 0.881445 | 0.001322 | 0.010577 | 0.105773 | 0.000881 |
| S9 | 0.881445 | 0.001322 | 0.010577 | 0.105773 | 0.000881 |
| S10 | 0.881445 | 0.001322 | 0.010577 | 0.105773 | 0.000881 |

**Table S5** Status of contamination (S_c_) of heavy metals

|  | Sc | | | | |
| --- | --- | --- | --- | --- | --- |
| **Sample points** | Cd | Cu | Fe | Pb | Zn |
| S1 | 3 | 0.0035 | 0.072 | 0.76 | 0.006 |
| S2 | 2.66667 | 0.0045 | 1.36 | 0.8 | 0.02 |
| S3 | 3.33333 | 0.005 | 0.092 | 8.8 | 0.0567 |
| S4 | 3.33333 | 0.005 | 0.032 | 0.4 | 0.1 |
| S5 | 3 | 0.005 | 0.036 | 0.4 | 0.003 |
| S6 | 2.33333 | 0.005 | 0.028 | 0.36 | 0.003 |
| S7 | 3 | 0.0045 | 0.028 | 0.36 | 0.003 |
| S8 | 2 | 0.0035 | 0.024 | 0.36 | 0.003 |
| S9 | 2.33333 | 0.003 | 0.036 | 0.32 | 0.003 |
| S10 | 1.66667 | 0.0025 | 0.024 | 0.24 | 0.003 |

**Table S6** Overall water quality O_w_

|  | Ow | | | | |
| --- | --- | --- | --- | --- | --- |
| **Sample points** | Cd (Ow) | Cu (Ow) | Fe (Ow) | Pb (Ow) | Zn (Ow) |
| S1 | 2.644336 | 4.63E-06 | 0.000762 | 0.080388 | 5.29E-06 |
| S2 | 2.350521 | 5.95E-06 | 0.014385 | 0.084619 | 1.76E-05 |
| S3 | 2.938152 | 6.61E-06 | 0.000973 | 0.930806 | 4.99E-05 |
| S4 | 2.938152 | 6.61E-06 | 0.000338 | 0.042309 | 8.81E-05 |
| S5 | 2.644336 | 6.61E-06 | 0.000381 | 0.042309 | 2.64E-06 |
| S6 | 2.056706 | 6.61E-06 | 0.000296 | 0.038078 | 2.64E-06 |
| S7 | 2.644336 | 5.95E-06 | 0.000296 | 0.038078 | 2.64E-06 |
| S8 | 1.762891 | 4.63E-06 | 0.000254 | 0.038078 | 2.64E-06 |
| S9 | 2.056706 | 3.97E-06 | 0.000381 | 0.033848 | 2.64E-06 |
| S10 | 1.469076 | 3.31E-06 | 0.000254 | 0.025386 | 2.64E-06 |

**Table S7** Average daily intake ADI

| **Sample points** | Cd | Cu | Fe | Pb | Zn | Cd |
| --- | --- | --- | --- | --- | --- | --- |
| S1 | 0.0003 | 0.0003 | 0.0003 | 0.0005 | 0.0006 | 0.0514 |
| S2 | 0.0003 | 0.0003 | 0.0003 | 0.0005 | 0.0063 | 0.0514 |
| S3 | 0.0003 | 0.0003 | 0.0003 | 0.0002 | 0.0063 | 0.0514 |
| S4 | 0.0003 | 0.0003 | 0.0003 | 0.0001 | 0.0023 | 0.0514 |
| S5 | 0.0003 | 0.0003 | 0.0003 | 0.0001 | 0.0014 | 0.0514 |
| S6 | 0.0003 | 0.0003 | 0.0003 | 0.0001 | 0.1260 | 0.0514 |
| S7 | 0.0000 | 0.0000 | 0.0000 | 0.0001 | 0.0000 | 0.0000 |
| S8 | 0.0003 | 0.0003 | 0.0003 | 0.0001 | 0.0023 | 0.0514 |
| S9 | 0.0000 | 0.0000 | 0.0000 | 0.0005 | 0.0000 | 0.0000 |
| S10 | 0.0003 | 0.0003 | 0.0003 | 0.0005 | 0.0043 | 0.0514 |
